# Investigation of Correlation between Communication Skills and Self-Reported Elder Mistreatment in Family Abuse

**DOI:** 10.1055/s-0044-1787300

**Published:** 2024-07-01

**Authors:** Sogra Zarei, Mohammad Esmaeilpour-Bandboni, Roya Mansour-Ghanaei, Iman Alizadeh

**Affiliations:** 1 Student Research Committee, Zeynab (P.B.U.H) School of Nursing and Midwifery, Guilan University of Medical Sciences, Rasht, Iran; 2 Zeynab (P.B.U.H) School of Nursing and Midwifery, Guilan University of Medical Sciences, Rasht, Iran; 3 Gastrointestinal and Liver Diseases Research Center, Guilan University of Medical Sciences, Rasht, Iran; 4 Department of English Language Teaching, School of Medicine, Guilan University of Medical Sciences, Rasht, Iran

**Keywords:** older adult, aged, elder abuse, communication, old age, elderly abuse, communication skills

## Abstract

**Introduction** In today's world, old age has become an important global phenomenon following the increase in life expectancy and the decrease in birth rates. Communication skills are an important requirement in old age. Changing role of the family and existing tensions, mental pressures, and modern life undermine the social position of the elderly and lead to abuse of the elderly by family members. The goal of the present study is to determine the relationship between communication skills and family self-reported domestic abuse among older adult in Iran.

**Materials and Methods** For this cross-sectional-analytical study, 153 elderly adult people admitted in hospitals of the Guilan province were randomly selected. The research instruments were the following questionnaires: demographic characteristics, abbreviated mental test (AMT), the Persian version of Domestic Elder Abuse Questionnaire, family mistreatment of the elderly (Heravy), and Queendom Communication Skill Test–Revise (QCSTR). The data were analyzed by SPSS software (version 22) using descriptive (frequency distribution tables, mean, and standard deviation [SD]) and analytical statistics (Mann–Whitney, Kruskal–Wallis, and Spearman's correlation tests) considering the significance level of 0.05.

**Results** A majority of the elderly were men (51%), were in the age group of 60 to 69 years (72.5%) and married (75.5%), did not hold high school diploma (88.8%), had four to five children (41.2%) with low income (75.9%), and suffered from chronic diseases (68.6%). The mean score of communication skills was 129.09 ± 12.60. The mean score of domestic elder abuse was 2.89 ± 3.97. Communication skills have a significant relationship with age and marital status, but not with sex, education level, income, and chronic disease. There is an inverse correlation between communication skills and domestic elder_abuse ( *p* < 0.001, *r* = −0.468).

**Conclusion** Communication skills are one of the influential factors of domestic violence. Therefore, to prevent or reduce the amount of violence, it is recommended that family members increase the communication skills of the elderly.

## Introduction

In this era, following the increase of life expectancy and decreasing birth rates, older age has become an important global phenomenon. 1 Human aging is an irreversible and irremediable process that is accompanied by morphological, functional, and biochemical changes in the human body. It is predicted that the percentage of world's population older than 65 years will double from 11 to 22% by 2050, so that 2 billion people will live 65 years or longer around the world. Approximately 400 million people will be ≥80 years by 2050. 2 According to a 2015 census in Iran, the population of elderly people older than 60 years comprises 9.2% of the total population of the country. 3 Aging is significantly associated with several negative outcomes, such as poor mental and physical health, functional and cognitive impairment, and bereavement. 4 During old age, people face a series of physical, mental, and social deprivations that they cope with. 5 Family is the smallest social unit but the most important supportive and pedagogical unit. 6 Changing role of the family and prevailing tensions, mental pressures, modern life, and the like deteriorate the social position of the elderly, leading to domestic abuse of the elderly. 7

According to the World Health Organization (WHO), elder abuse is defined as “a single or repeated act” where a relationship lacks proper respect and eventually causes injury or harm to an elderly person. 8 Elder abuse is a serious human rights violation and a global threat that requires immediate action. It is also a major public health problem leading to serious health consequences for victims, including increased risk of cancer, mortality, and hospitalization, and has a negative impact on families and society. 9 10

The global prevalence of elder abuse in social environments is 15.7%, or approximately one in six elderly people. 11 In Iran, the most common types of elder abuse are as follows: emotional (30.7%), psychological (25.4%), financial (19.7%), physical (13.1%), neglect (25.1%), and abandonment (11.7%), with an overall 56.4% prevalence in the general population. 12 Researchers have found that elder abuse is underreported, which can be a function of fear or embarrassment, especially if older people are abused by family members. Elder abuse can cause long-term physical and mental harm as well as social damage, including homelessness. 11

Old age is a sensitive period of human life, and it is necessary to pay attention to the issues and needs of this time. The increase in the number of elderly people leads to the growth of their problems, and identifying the real needs of seniors helps planners to take appropriate actions for their requirements, including communication skills. 13 14 Some of these communication skills fade away with increasing age, physical problems and disabilities, as well as staying away from the society. 15

Communication skills or interaction with others is one of the most important and valuable components of every person's life, which has received attention in recent years 16 so that it is a basic and challenging concern for success in human life. 17 Today, correct communication is considered one of the most essential needs for progress and success in both personal and social life. 18

Communication is one of the basic elements of human performance as well as the foundation of interpersonal relationships. Interpersonal relationships start and develop through communication, and communication has an impact on the quality of interpersonal relationships. 19 Communication skills are a set of potential and actual abilities of a person through which an acceptable and informative behavior can be achieved until a level of emotional relationship is reached. These skills are so important that their absence could lead to loneliness and stress in a person, decrease self-confidence and self-respect, and lower academic and work achievement. 20 21 22

Communication skills include the subskills of understanding verbal and nonverbal messages, listening, insight into the communication process, regulating emotions, and assertiveness in communication. 22 Understanding verbal and nonverbal messages is considered the ability to send and receive clear communication messages, which is referred to as message comprehension skills. 17

Listening has been described as a multistep process that involves techniques such as commenting, preparing, and setting appropriate questions, repeating and summarizing to express full understanding and confirm what has been said. It also includes maintaining eye contact, using nonverbal gestures such as nodding or smiling, and interrupting the speaker. The purpose of active listening is to develop a clear understanding of_the speaker's concern and also to clearly express the listener's interest in speaker's message. 23

Emotion regulation is broadly defined as the capacity to manage one's emotional responses, which involves strategies to increase, maintain, or decrease the intensity, duration, and trajectory of positive and negative emotions. Learning to regulate emotions is a key social emotional skill that allows for flexibility in motivational situations. 24

Alberti and Emmons define assertiveness as a behavior that enables people to act in their own interests, defend themselves without undue anxiety, express their feelings honestly, or exercise their rights without violating others' rights. Assertiveness is often confused with aggression. However, aggression involves misrepresenting opinions and feelings in a way that ignores the rights of others. 25 The study of Yarinasab and Amini found communication skills to be effective in domestic violence against women. 26 The study of Ansari et al showed an inverse relationship between communication skills and the degree of physical violence. 27 The research by Rezaei et al indicated the utility of communication skills training in reducing violence against nurses. 28 The number of older adult_people and the prevalence of elder abuse have an upward trend. 12 Elder mistreatment is always associated with numerous physical, emotional, and family problems, and recognizing the factors affecting this destructive behavior can be helpful in designing programs to prevent the spread of elder abuse. There has been no study in relation to elder mistreatment and its relationship with communication skills in Guilan province, the province with the highest number of seniors in the country. Therefore, the present study was conducted with the aim of determining the correlation between communication skills and elder abuse in Guilan province. Recognizing the prevalence of this problem and its related factors will be useful for nursing researchers and geriatric health policymakers to formulate prevention programs and relevant solutions.

## Materials and Methods

The current cross-sectional analytical study was conducted in 2021 to 2022 with the aim of determining the correlation between communication skills and family mistreatment of seniors admitted to teaching hospitals of Guilan province in northern Iran. In this research, 153 elderly people ≥60 years were randomly selected from six governmental hospitals by proportional stratified random sampling.

The following inclusion criteria were considered: age ≥60 years, willingness to participate in the research (voluntary consent), and not suffering from deafness, blindness, aphasia, and psychological disorders (≥8 score on Persian version of abbreviated mental test [AMT]). Reluctance and unwillingness to continue to cooperate during the research were the exclusion criteria.

The published questionnaires were completed face to face for each person individually and with respect for his or her privacy. We read aloud the questionnaires to the senior participant_and the questionnaire items were filled based on the opinion and choice of the elderly. There were four questionnaires: demographic characteristics, AMT, the Persian version of Domestic Elder Abuse Questionnaire that was published in Heravi's study, and Queendom Communication Skill Test–Revise (QCSTR).

AMT has 10 items, each with 1 point. The reliability and validity of the original version 29 as well as the Persian version of the mentioned questionnaire have been confirmed. A score of ≥8 in this test meant the absence of cognitive impairment, 30 and in case of a score of greater than 8, the other sections of the questionnaire were completed.

The second part included the demographic information of the seniors such as age, gender, education, income level, number of children, and whether or not they were suffering from chronic diseases.

The third part was CSTR prepared by Queendom in 2004 to measure the communication skills of adults. It has 34 items that describe the communication skills in five subscales. The ability to receive and send messages (9 questions), emotional control (9 questions), listening skills (6 questions), insight into the communication process (5 questions), and assertive communication (5 questions).

The score range for each subject is 34 to 170. The research unit determines his or her degree of compliance with current circumstances on a 5-point Likert scale from 1 for never to 5 for forever. A higher score indicates better communication skills. Some statements are scored in the reverse. If the choice of always for other items means maximum score of 5, in reverse items, choosing the same answer leads to the minimum score of 1. The validity of this questionnaire in Iran has been checked and confirmed by Hosseinchari and Fadakar, and the internal consistency of the questionnaire items was reported as 0.69 using Cronbach's α method. 31 In our study, Cronbach's α value was calculated as 0.88.

The fourth part of the instrument was the standard questionnaire examining *domestic* elder mistreatment/abuse in the family. This questionnaire has 49 items in eight subscales: emotional, care and financial neglect, psychological abuse, deprivation of authority, physical and financial abuse, and abandonment. The items of the tool have the following options: yes, no, and not relevant. The obtained scores are in the range of 0 to 100, and a higher score indicates more severe symptoms of domestic elder abuse / family mistreatment. Therefore, a score of 100 indicates maximum abuse and 0 means the absence of evidence of mistreatment/abuse. The score 0 corresponds to no option and 1 to the yes option, and the not relevant option means no points and is ignored when calculating the score. This measurement abuse tool has been compiled and validated according to the findings of a qualitative study on abused elderly people in Iran according to cultural features of the Iranian society with confirmed face, content, and structure validity. Cronbach's α coefficient for reliability of this tool has been 0.97. 32 For data analysis, descriptive statistics (frequency distribution tables, mean, standard deviation, and graphs) as well as inferential statistics (Mann–Whitney, Kruskal–Wallis, and Spearman's correlation tests) methods were used in SPSS 22 software with a significance level of *p* < 0.05.

## Results

Out of 153 senior people participating in this study, 51% were men, and the mean age of the participants was 66.29 ± 4.93 years. The minimum and maximum ages were 60 and 80 years, respectively. In all, 75.5% of the participants were married. The majority of the participants (88.8%) did not hold a high school diploma, 41.2% of the elderly had four to five children, 75.9% had low income, and 68.6% had a chronic disease ( Table 1 ).

**Table 1:** Frequency distribution and percentage based on demographic characteristics

Variables		Frequency (%)	Variables		Frequency (%)
Sex	Men	78 (51)	Children (y)	1 ≤	9 (5.9)
	Women	75 (49)		2–3	41 (26.4)
				4–5	63 (41.2)
Age group (y)	60–69 70	111 (72.5) 42 (27.5)		6 ≥	40 (26.1)
Education	High school	136 (88.8)	Income	Low	116 (75.9)
	Diploma	14 (9.2)		Middle	25 (16.3)
				High	12 (7.8)
	Collegiate	3 (2)	
Chronic disease	Yes	105 (68.6)	Marital status	Married Lost wife	115 (75.5) 38 (24.5)
	No	48 (31.4)			

The mean and SD of communication skills in the study population was 129.09 ± 12.60 with respective minimum and maximum of 90 and 154. These values for domestic abuse were reported as 2.89 ± 3.97 with the lowest and highest values of 0 and 22, respectively. Communication skills had a significant relationship with age and marital status. In other words, communication skills decrease with age, and married people have better communication skills. There was no significant relationship between communication skills and education and income. These skills were different according to the number of children; in other words, communication skills had a significant relationship with the lower number of children ( *p* < 0.05; Table 2 ).

**Table 2:** Relationship between demographic variables and communication skills

Variables		Frequency	Average	*K* or *Z*	*p* value
Sex	Woman	75	79.84	−0.77 a	0.433
	Men	78	74.27		
Age group (y)	60–69	111	88.63	−5.28	0.000
	70	42	46.26		
Marital status	Married	115	82.37	−2.60	0.009
	Lost wife	38	60.76		
Chronic disease	No Yes	48 105	83.23 73.39	−1.28	0.20
Education	High school	136	76.02	1.29 b	0.52
	Diploma	14	89.00		
	Collegiate	3	65.50		
Children (y)	1≤	9	84.89	4.62	0.20
	2–3 4–5	41 63	85.64 77.67		
	6≥	40	65.34		
Income	Low	116	74.10	2.23	0.31
	Middle	25	88.78		
	High	12	80.46		

a Mann–Whitney *U* test.

b Kruskal–Wallis test.

Based on statistical tests, there was a significant and inverse relationship between communication skills and areas of domestic elder abuse. In other words, the domestic elder abuse_areas decrease with the increase in communication skills. The highest and lowest correlation was related to psychological misbehavior abuse ( *r* = −0.367) and rejection ( *r* = −0.173), respectively ( Table 3 ).

**Table 3:** Correlation between communication skills and areas of family mistreatment of the elderly

Variable 1	Variable 2	Spearman's test	*p* value
Communication skills	Elderly mistreatment in the family	−0/468	0/000
	Emotional abuse	−0/179	0/027
	Caring neglect	−0/335	0/000
	Financial neglect	−0/312	0/000
	Deprivation of authority	−0/329	0/000
	Psychological abuse	−0/367	0/000
	Physical abuse	−0/198	0/014
	Financial misconduct	−0/245	0/001
	Rejection	−0/173	0/032
	Communication skills	−0/468	0/000
	Insight into the communication process	−0/323	0/000
Elder abuse	Ability to receive and send messages	−0/331	0/000
	Listening skill	−0/284	0/000
	Regulating emotions	−0/382	0/000
	Assertiveness in communication	−0/364	0/000

The statistical tests show that there is an inverse correlation between *domestic abuse* and areas of communication skills. The highest correlation of domestic abuse_was observed with the domain related to emotional control ability of communication skills ( *r* = −0.382), and listening skills ( *r* = −0.284) had the lowest correlation among communication skills ( Table 3 ).

## Discussion

The purpose of this research was to study the communication skills and examine their relationship with domestic elder abuse_among seniors admitted to educational hospitals of Guilan province in northern Iran. The findings showed that the mean and SD of the communication skills of the hospitalized elderly is 129.09 ± 12.60. In the study of Hosseini Ramaghani et al, the mean and SD of communication skill scores of the senior participants was 108.30 ± 34.54, 15 which was 114.51 ± 9.93 in Mojadam et al's study. 13 These results indicate that the communication skills of the elderly participating in this study are at a favorable level (129.09 ± 12.60). This difference can be due to diverse cultural conditions of different regions.

In this study, there was a significant correlation between communication skills of the elderly with age and marital status. In relation to age, the results of this study are consistent with those of Yusefi et al, 14 Mojadam et al, 13 and Shakerinia. 33 In other words, the communication skills of the elderly decrease as their age progresses. The results of the study by Khatib Zanjani and Moharreri 18 and Hadi et al, 34 which were respectively conducted among nurses and university professors, were not consistent with the findings of the present study. The reason for this inconsistency can be the difference in research units and measurement tools. With increasing age, some of these communication skills decrease due to physical problems and disabilities caused by old age and distance from society. 15

In relation to the marital status, the present study showed that married elderly have better communication skills than those who have lost their spouses. The results of our research are in line with the findings of Yusefi et al 14 and Qolizadeh et al 35 but not with the study of Ebadi 36 and Khatib Zanjani and Moharreri. 18 This inconsistency can be related to the difference in research units as well as the measurement tools. The study of Ebadi and Khatib Zanjani and Moharreri were conducted among nursing students and nurses, respectively. Besides, these two studies were conducted using researcher-developed questionnaires. As the seniors grow older, they need a companion who listens to what they say. In other words, being married in old age means having a companion for conversation, and the difference in age between elders and nurses could account for the inconsistent findings of these two studies.

In this study, no significant relationship was observed between communication skills of the elderly and gender, education level, and income. Regarding gender, our results are in line with the study of Khatib Zanjani and Moharreri 18 and Gheirati et al. 37 The findings of the present study are not in line with those of Yusefi et al, 14 Mojadam et al, 13 and Zhou et al. 38 The reason for this difference could be difference in cultural conditions, research units, and measurement instruments. Also, in Mojadam et al's study, the number of men is higher (79.3%); therefore, men's skills have been higher than those of women.

Regarding the level of education, the results of the present study are in line with the findings of Coats et al 39 and Khatib Zanjani and Moharreri. 18 It is not in agreement with the study of Yusefi et al 14 and Qolizadeh et al, 35 which could be due to the difference in research units, measurement instruments, and sample size.

Regarding the income level, the results of this study are consistent with the study of Khatib Zanjani and Moharreri. 18

Elder abuse has been internationally recognized as a widespread and serious problem that requires urgent attention of health care systems, social welfare agencies, policymakers, and the general public. 9 In this study, an inverse and significant correlation was observed between communication skills and areas of domestic abuse_of the elderly so that with the increase of communication skills, the areas of elder abuse_were reduced. The results of the present research are in line with those of Rezaei et al, 40 Yarinasab and Amini, 26 Rahimi et al, 41 Delsuz et al, 42 and Alargic et al. 43

The present research showed an inverse and significant correlation between the variable of elder domestic abuse_and the areas of communication skills. Ansari et al's study was conducted with the aim of assessing the effect of women's communication skills on domestic violence by husbands, which showed that higher communication skills led to a reduction in violent behavior by spouses. Among communication skills, verbal skill is the most important to reduce both physical and nonphysical violence. These results are in line with the findings of the present study. 27 Among the areas of communication skills, the least important correlation was found between listening skill and the most important with communication with assertiveness. An assertive person has control over his or her life and all aspects of it, including work, interpersonal relationships, intimate relationships, parenting, etc. These people know how to take care of themselves, give constructive criticism, and also be criticized without feeling offended. In addition, such a person is a good speaker. Assertive communication increases self-confidence and reduces anxiety. 43 All these factors can help reduce mistreatment of the elderly.

## Conclusion

As we know, domestic abuse has short- and long-term harmful effects on the elderly. Communication skills have an inverse relationship with the prevalence of family violence. Nursing researchers in the field of geriatrics should pay more attention to elder abuse. Elderly victims of violence should be given social support and the prevalence of elder abuse should be reduced through communication skills training.

## Limitations

Our study may have the following three limitations:

- The study is observational in nature and at a single point in time. Therefore, inherent limitations of observational studies exist. Additionally, the findings do not reflect longitudinal changes over time.
- The nature of the captured data can only offer association findings and no causation can be inferred from this study.
- There may be other confounders to the endpoints measured that we did not know about and therefore did not account for.
